# Disruption of Placental Immune Privilege by Complications of Pregnancy: Consequences for Maternal and Child Health

**DOI:** 10.3390/ijms27209247

**Published:** 2026-10-18

**Authors:** Wendy Kisaí Mixtega-Ruiz, Mariela Contreras Escamilla, Rosalba Sevilla-Montoya, Andrea Olmos-Ortiz, Verónica Zaga-Clavellina, Diana Catalina Castro-Rodríguez

**Affiliations:** 1 Doctorado en Ciencias Biológicas, Centro Tlaxcala de Biología de la Conducta, Tlaxcala C.P. 90070, Mexico; wenki_96@hotmail.com; 2 Departamento de Inmunobioquímica, Instituto Nacional de Perinatología, Isidro Espinosa de los Reyes, Mexico City C.P. 11000, Mexico; andrea.olmos@inper.edu.mx; 3 Departamento de Investigación y Bioterio, Instituto Nacional de Ciencias Médicas y Nutrición Salvador Zubirán, Mexico City C.P. 14080, Mexico; mariela.contrerase@incmnsz.mx; 4 Coordinación de Genética y Genómica Humana Instituto Nacional de Perinatología, Mexico City C.P. 11000, Mexico; rosalba.sevilla@inper.edu.mx; 5 Dirección de Investigación, Instituto Nacional de Perinatología, Isidro Espinosa de los Reyes, Mexico City C.P. 11000, Mexico; veronica.zaga@inper.gob.mx

**Keywords:** maternal chronic conditions, placental, immune privilege, offspring

## Abstract

Maternal chronic conditions, including obesity, diabetes, hypertensive disorders, and autoimmune diseases, promote a systemic proinflammatory environment characterized by oxidative stress and chronic inflammation. These alterations lead to reduced expressions of human leukocyte antigen-G (HLA-G) and programmed death-ligand 1 (PD-L1), decreased regulatory T (Treg)cell activity, and increased T helper (Th)1/Th17 responses, disrupting placental immune privilege and resulting in increased production of proinflammatory cytokines, oxidative stress, trophoblastic dysfunction, and placental senescence. This change in immune privilege contributes to fetal growth restriction, preterm birth, congenital anomalies, and long-term cardiometabolic programming in offspring. Understanding the mechanisms underlying placental immune dysregulation may facilitate the development of targeted therapeutic strategies aimed at restoring immune tolerance and reducing inflammatory and hypoxic signaling pathways to improve maternal and neonatal outcomes. This review summarizes the current state of the field of placental immunological privilege and how maternal chronic conditions like obesity, diabetes, hypertensive disorders, and autoimmune diseases can disrupt it and its possible consequences on the progeny.

## 1. Introduction

Pregnancy is a complex physiological process in which the mother undergoes body changes to establish a specialized systemic environment that supports fetal growth. Since the discovery that both peripheral innate and adaptive immune human cells express sex hormone receptors [1,2], it has been suggested that changes in hormone secretion during the menstrual cycle create an optimal condition for successful conception. Immune cells in the endometrium comprise heterogeneous populations that include decidual Natural Killer (dNK) cell subtypes, decidual macrophages (dMϕs), cluster of differentiation 4-positive (CD4+) and 8-positive (CD8+) T cells, dendritic cells (DCs), B cells, and mast cells [3]. It has been reported that uterine Natural Killer (uNK) cells, macrophages, DCs, and T cells are the predominant cells that are increased in the secretory phase, coincident with the onset of decidualization and early in pregnancy [3,4,5]. Although their numbers grow, each immune cell population in the uterine microenvironment exhibits unique phenotypic and functional characteristics. During early stages of pregnancy, specialized immune subsets engage in intense interaction with extravellous trophoblasts (EVTs) to promote tissue remodeling, angiogenesis, and immune tolerance. If these mechanisms are disrupted by pregnancy complications such as preeclampsia (PE), gestational diabetes mellitus (GDM), and obesity-related metabolic dysfunction, they can compromise the composition and activation of immune cells at the maternal-fetal interface, affecting placental function and fetal development, and leading to long-term health consequences for the offspring. Understanding how placental immune privilege is established, maintained, and perturbed is therefore critical for identifying therapeutic strategies that enhance maternal and neonatal outcomes. This review summarizes current understanding of the placental immunological privilege and its disruption in pregnancy disorders, as well as clinical consequences in offspring and current pharmacological therapy. Here, we present a comprehensive and non-systematic literature search that was conducted across electronic databases including PubMed, Scopus, and Google Scholar, from 2015 up to the current year. Studies were selected if they directly addressed placental immunological disruption by various types of chronic diseases, their impact on progeny, and how the therapies modified it, or provided historical context.

## 2. Placental Immune Privilege: Mechanisms and Regulation

In early human pregnancy, uNK cells account for approximately 70% of total lymphocytes, and their proportion gradually diminishes as gestation progresses [6,7]. Currently, six major subsets of innate lymphoid cells (ILCs) have been described and proposed to be present in the human uterus early in pregnancy. These include decidual natural killer 1-3 (dNK1-3) cells, decidual proliferating NK (dNKp) cells, decidual group 3 innate lymphoid cells (dILC3), and peripheral blood NK (pbNK) cells [8]. In particular, dNK2 and dNK3 cells produce higher levels of X-C motif chemokine ligand 1 (XCL1), macrophage inflammatory protein (MIP)-1α (also termed CCL3), MIP-1β (also termed CCL4), and granulocyte-macrophage colony-stimulating factor (GM-CSF), as well as interferon gamma (INF-γ) and cluster of differentiation 107a (CD107), the latter two being expressed at levels comparable to those in pbNK cells. Interestingly, in the absence of HLA-C molecules but under Phorbol 12-myristate 13-acetate (PMA)/Ionomicine stimulation, dNK2 and dNK3 cells produced higher levels of XCL1 and CCL4 than pbNK cells [8]. These findings suggest that these populations play distinct roles during gestation. If there is one clear now, it is that the phenotype of dNK cells represents a population with limited cytolytic activity compared to pbNK [8].

NK cells establish direct contact with EVTs cells through the recognition of HLA-C, HLA-G, and HLA-E molecules expressed on the trophoblast surface, via killer immunoglobulin-like receptors (KIRs), leukocyte immunoglobulin-like receptor B1 (LILRB1), and natural killer group 2 (NKG2) receptors, respectively [9]. In vitro activation of KIR2DS1 by its ligand HLA-C2 induces GM-CSF production, which promotes the migration of JEG-3 trophoblast cells. Furthermore, a close association between GM-CSF, NK cells, and trophoblast cells has been observed in the maternal decidua, supporting these findings [9]. In a recent study, trophoblast organoids exposed to cytokines such as XCL1, CSF1, CSF2, and CCL5 promoted EVT differentiation and upregulated gene pathways involved with cell invasion and fusion [10]. Notably, these genes were also observed in ETVs from primary tissue samples, suggesting comparable roles in vitro and in vivo [10].

On the other hand, macrophages represent the second most predominant immune cell population in decidual tissue. Although increased evidence has shown that M1 macrophages are present during early pregnancy [11,12,13]. Isolated leukocytes from the secretory phase of human endometrium exhibit both M1 and M2 macrophage phenotypes, with the M2 phenotype being predominant. These M2 macrophages produce IL-6, CXCL8, CCL4, CCL2, and vascular endothelial growth factor (VEGF) under basal conditions [14]. Interestingly, in vitro assays demonstrate that the interaction with trophoblasts and macrophages enhances the secretion of IL-6, TGF-β, IFN-γ, and IL-10 by macrophage cells, while promoting the migration and invasion of trophoblasts [15]. Even though the M2 phenotype is related to an anti-inflammatory response, M2 macrophages are divided into M2a, M2b, M2c, and M2d subtypes, some of which are involved in triggering a pro-inflammatory and angiogenic phenotype [12]. Mani et al. demonstrated that M1 macrophages are abundant in the endometrium during the implantation window. Following trophoblast invasion of the decidua, a regulatory shift toward an M2-like phenotype could occur during the first trimester [16].

Regulatory T (Treg) cells are also present during early pregnancy. These cells are derived from T CD4+ cells and express the transcription factor Foxp3. In the uterus, their phenotype and expansion are regulated by seminal fluid [17,18]. Furthermore, in preclinical models using mice, the removal of Treg cells following mating leads to implantation failure [19]. Therefore, these cells are essential during the peri-implantation window. The interaction of signals between the EVT and immune cells plays a crucial role in establishing immune tolerance and ensuring optimal implantation. Over the past decade, there has been growing interest in exosome-mediated cellular communication. Exosomes are nanoscale extracellular vesicles (between 30 and 200 nm in diameter) surrounded by a lipid bilayer and capable of transporting various biomolecules, including mRNA, miRNA, lipids, and proteins. Exosomes are isolated from human primary trophoblast-derived or Sw71 EVT-like cells expressed CD63+, HLA-G, HLA-C, and HLA-E [20,21]. Moreover, HLA-E-bearing exosomes derived from EVT appear to induce IFN-γ and VEGFα secretion by dNK cells through an mTORC1 phosphorylation-dependent mechanism [21]. This signaling pathway seems to be essential for promoting angiogenesis, trophoblast growth, and regulating Th17 responses [21,22].

Currently, three subsets of decidual macrophages have been identified based on the expression of C-C chemokine receptor type 2 (CCR2) and integrinα-X (CD11c): macrophages with proinflammatory characteristics (CCR2 + CD11chigh) resembling the M1 phenotype, those with anti-inflammatory characteristics (CCR2-CD11chigh) resembling the M2 phenotype, and a third subset distributed throughout the decidua (CCR2-CD11clow) [13]. Whereas in the placental tissue, Hofbauer cells and the M3 phenotype predominate [11]. The CCR2-CD11clow subset is abundantly distributed within the decidua parietalis, a region where EVTs are absent [11]. Vondra et al. described macrophages within the decidua parietalis exhibiting enhanced phagocytic activity and the capacity to induce T-cell proliferation, suggesting a role as patrolling macrophages poised to respond rapidly to infections [23]. These macrophages exhibit gene expression signatures associated with the CD11low phenotype, as previously reported [13,23]. In contrast, CD11-high macrophages present in the decidua basalis secrete high levels of CXC-motif chemokine ligand 1 (CXCL1), CXC-motif chemokine ligand 5 (CXCL5), macrophage-colony stimulating factor (M-CSF), and IL-10; moreover, they also induce Treg cell expansion [23].

Furthermore, it has been reported that macrophages can internalize exosomes derived from trophoblast cells, resulting in an increased proportion of M2-like macrophages [24]. In addition, macrophages treated with trophoblast-derived exosomes show inhibited T-cell proliferation [25]. Furthermore, histone deacetylase 8 (HDAC8) may contribute to maintaining the M2 phenotype in macrophages by activating the ERK signaling pathway [26]. Taken together, these signals may exert control over macrophage function, promoting an immunosuppressive environment conducive to a successful pregnancy. In addition, the presence of inhibitory molecules may contribute to creating an immunosuppressive environment. The major pathways involved are programmed cell death-1 and its ligand (PD1/PDL-1) and T-lymphocyte-associated protein-4 (CTLA4) [27]. These molecules are expressed on various immune cells in the maternal-fetal interface, and the goal is to regulate the magnitude of immune responses, thereby favoring placental development [27,28].

Additional inhibitory molecules are also employed by immune cells, such as T-cell immunoglobulin domain and mucin domain-containing molecule-3 (Tim-3). Tim-3 is a type I transmembrane glycoprotein expressed on dMϕs, decidual T cells (both CD4+ and CD8+ T cell subsets), decidual DCs, and placental Mϕs [29]. In vitro assays, which mimic the process of stromal cell decidualization, show that Galectin-9 (Gal-9) on endometrial stromal cells increases as decidualization progresses. This process is further enhanced by the presence of dNK cells [29,30]. Moreover, it has been reported that ubiquitin C-terminal hydrolase L1, a deubiquitinating enzyme, promotes decidualization and favors dNK cell recruitment through activation of the JAK2/STAT3 signaling pathway [31]. Later in pregnancy, engagement between Gal-9 on EVTs and Tim-3 on dNK cells promotes higher IL-4 and lower TNF-α production, maintaining their low cytotoxicity activity [32]. In addition, Huang et al. demonstrated that upregulation of miR-30e expression in dNK cells suppressed their cytotoxicity and enhanced angiogenic functions by increasing the expression of IL-4, IL-10, VEGF, angiopoietin 2, and placental growth factor [33]. However, whether Tim-3 interactions regulate miR-30e expression remains to be elucidated. Furthermore, Tim-3+ dMϕs induce CD4+ T-cell polarization toward Th2 and Treg phenotypes [34]. In turn, Treg cells form a regulatory loop in response to IL-27 produced by EVTs, leading to increased IL-10 production, suppression of effector T-cell proliferation, and induction of M2-like macrophage polarization. Altogether, these interactions contribute to maintaining an anti-inflammatory status [30,35,36].

In placental tissue from term non-laboring mothers, activated T cells were the most abundant population, followed by NK cells, Mϕs, monocytes (Mo), and B cells [37]. During term labor, the proportion of activated T cells—such as CD8+ cells that express granzyme B and perforin—increases significantly; these are two proinflammatory mediators that contribute to the inflammatory processes associated with labor [38]. Another important point is that this increase is accompanied by a shift toward the M1 macrophage phenotype, with higher expression of NF-kB1 throughout term labor [37]. The process of labor is a coordinated event primarily driven by smooth muscle cells (SMCs) of the myometrium, whose main role is to orchestrate uterine contractions that expel the fetus from the uterine cavity. These contractions are accompanied by an inflammatory cascade involving cytokines and chemokines such as IL-1β, IL-6, IL-8, and monocyte chemotactic protein-1 (MCP-1, also termed CCL2), produced by SMCs and leukocytes [39]. During spontaneous term labor, monocytes and macrophages are the most abundant immune cells found in myometrial tissue and are among the primary contributors to the production of inflammatory mediators [40]. Furthermore, in vitro assays suggest that inflammatory mediators produced by Mo may enhance myocyte contractility (Figure 1), establishing a positive feedback loop with prostaglandin F2α (PGF2α) during the labor process [39,40,41].

## 3. Chronic Diseases as Disruptors of Placental Immune Privilege

Placental immune privilege constitutes a fundamental biological mechanism for successful pregnancy, as it allows the coexistence of semi-allogeneic fetal tissues within an immunologically competent maternal environment. Far from representing a state of generalized immunosuppression, this immune privilege is the result of a complex network of cellular and molecular mechanisms that finely regulate the immune response at the maternal-fetal interface. Prominent among these mechanisms are the expression of immunomodulatory molecules by the extravillous trophoblast, particularly Human Leukocyte Antigen E and G (HLA-E, HLA-G) and Programmed Cell Death Ligand 1 (PD-L1), the expansion of regulatory T cells (Tregs), the polarization of decidual macrophages toward a tolerogenic M2 phenotype, the specialized activity of uterine NK (uNK) cells, and the local production of anti-inflammatory cytokines such as IL-10 and TGF-β [42,43,44,45].

However, the stability of this immunological microenvironment largely depends on the maternal physiological state. Over the last few decades, accumulating evidence has demonstrated that chronic diseases such as obesity, diabetes mellitus, and hypertensive disorders during pregnancy can significantly alter these tolerance mechanisms, thereby compromising placental immune privilege and promoting obstetric complications.

Maternal obesity is currently one of the primary factors threatening placental immune homeostasis. This condition is characterized by a state of chronic low-grade systemic inflammation associated with adipose tissue expansion, proinflammatory macrophage infiltration, and increased production of cytokines such as TNF-α, IL-6, and IL-1β [46]. During pregnancy, these inflammatory signals reach the placenta and modify its immunological function. Various studies have shown that placentas from women with obesity exhibit increased macrophage infiltration, higher expression of inflammatory mediators, and sustained activation of signaling pathways associated with NF-κB and oxidative stress [46,47]. Consequently, the trophoblast’s ability to maintain a tolerogenic environment is impaired, favoring an inflammatory profile that can affect both placental development and fetal health [48].

One of the most relevant mechanisms through which obesity compromises placental immune privilege is the disruption of the balance between immunoregulatory cells and proinflammatory effector cells at the maternal-fetal interface [49,50]. The chronic inflammation associated with obesity favors the functional reduction in regulatory T cells (Tregs) and promotes inflammatory responses mediated by Th1 and Th17 lymphocytes, disrupting the tolerance mechanisms necessary for pregnancy maintenance [51,52].

Pregestational diabetes mellitus and gestational diabetes constitute another major group of metabolic conditions capable of altering various mechanisms involved in maintaining maternal-fetal immune tolerance. Chronic exposure to a hyperglycemic environment induces significant changes in cellular metabolism, including mitochondrial dysfunction, alterations in cellular bioenergetics, and an increase in the production of reactive oxygen species (ROS) [53,54]. Multiple studies have shown that placentas from pregnancies complicated by diabetes present an increase in oxidative stress markers, accompanied by higher production of proinflammatory cytokines such as TNF-α, IL-32, and IL-6, as well as the activation of NF-κB-dependent inflammatory signaling pathways including inflammasome activation [55,56,57,58]. Together, these alterations favor the establishment of an inflammatory microenvironment that can compromise essential functions of both the trophoblast and the immune cells present at the maternal-fetal interface, thereby affecting the mechanisms that contribute to establishing and maintaining placental immune privilege.

Beyond its inflammatory effects, hyperglycemia can also alter molecular mechanisms directly involved in immune tolerance. Experimental studies suggest that elevated glucose concentrations modify the expression of immunoregulatory molecules in the trophoblast, including HLA-G and various inhibitory receptors involved in communication with maternal NK cells and T lymphocytes [59,60]. Simultaneously, diabetes-induced oxidative stress favors processes of cellular apoptosis and senescence that increase the release of damage-associated molecular patterns (DAMPs), which activate pattern recognition receptors on local immune cells. As a result, a vicious cycle is established where inflammation promotes tissue damage and tissue damage amplifies inflammation, progressively eroding the mechanisms that sustain the immune privilege of the placenta.

Chronic arterial hypertension and hypertensive disorders of pregnancy constitute another paradigmatic example of disrupted placental immunotolerance. Preeclampsia is currently considered a disease characterized by a combination of deficient placentation, oxidative stress, and immune dysregulation [61]. During a normal pregnancy, uterine NK cells interact with the extravillous trophoblast to promote spiral artery remodeling and ensure adequate placental perfusion. These interactions heavily depend on the recognition of HLA-C, HLA-E, and HLA-G molecules expressed by the trophoblast [60,62]. However, alterations in these communication pathways can compromise trophoblast invasion and result in insufficient vascular remodeling.

The resulting hypoperfusion generates repeated episodes of hypoxia and reperfusion, which increase the production of reactive oxygen species and trigger an intense inflammatory response. In this context, an increased production of TNF-α, IL-6, IL-1β, and anti-angiogenic factors such as sFlt-1 is observed, contributing to the systemic endothelial damage characteristic of preeclampsia [61,63,64,65].

Simultaneously, several studies have described a reduction in the frequency of Treg cells and an increase in Th17 cells in women with preeclampsia, reinforcing the hypothesis that the loss of immune tolerance actively participates in the pathophysiology of the disease [66].

Despite their etiological differences, obesity, diabetes, and hypertension share common pathophysiological mechanisms that converge on the alteration of placental immune privilege (Figure 2). Notable among these are chronic inflammation, oxidative stress, inflammasome activation, endothelial dysfunction, altered macrophage polarization, and the loss of balance between immunoregulatory and effector cells. These processes promote persistent activation of inflammatory signaling pathways, particularly those mediated by Toll-like receptors (TLRs) and NF-κB, reducing the placenta’s capacity to maintain a proper tolerogenic environment [42]. In light of the above, it is important to emphasize that placental immune privilege is a dynamic state that is highly sensitive to the mother’s metabolic and inflammatory conditions, given that chronic diseases not only alter the placenta’s immune function but also modify essential processes of vascular development, tissue remodeling, and maternal-fetal communication. Understanding these mechanisms by which immune tolerance may be lost could open new opportunities for the development of interventions aimed at preserving the placenta’s immune homeostasis and improving maternal and fetal outcomes.

## 4. Effects on Progeny

Chronic maternal conditions such as obesity, diabetes (including gestational diabetes), and hypertensive disorders like preeclampsia create a biological milieu that differs markedly from the tightly orchestrated immune environment required for normal placentation. In a healthy pregnancy, the maternal immune system is not simply “suppressed,” but rather recalibrated to maintain fetal tolerance while preserving the host’s defenses. When a pathological condition arises during pregnancy, maintaining that balance becomes more difficult, which alters the intrauterine environment and triggers a cascade of effects in which the placenta is subjected to persistent inflammatory and metabolic stress, and the maternal-fetal interface may evolve toward a more activated and less tolerant immune profile, leading to fetal rejection and posing a risk to the health of the maternal-fetal unit. Recent work underscores that changes in local immune cell populations—particularly decidual macrophages and fetal macrophages (Hofbauer cells)—may accompany, or actively contribute to, abnormal placental development and function [67].

Low-grade systemic inflammation (as seen in obesity and diabetes), autoimmunity (e.g., systemic lupus erythematosus and rheumatoid arthritis), and dysbiosis can amplify cytokine and chemokine cascades (including IL-6, TNF, and CXCL-family chemokines), impair extravillous trophoblast invasion, and blunt uteroplacental perfusion; pathways tightly linked to both fetal growth failure and the timing of parturition. Emerging clinical and translational evidence supports this biological mechanism. On the one hand, inflammatory markers in the early stages of pregnancy are associated with preterm birth and adverse perinatal outcomes in patients with pregestational diabetes. On the other hand, various complementary studies provide a clearer picture of how inflammatory and microbial factors contribute to low birth weight in different contexts (Table 1). Taken together, these findings point to a continuum in which immune imbalance acts as the link between maternal chronic diseases and adverse perinatal outcomes [68].

### 4.1. Intrauterine Growth Restriction (IUGR)/Fetal Growth Restriction (FGR)

In contemporary obstetric practice, American College of Obstetricians and Gynecologists favors the term fetal growth restriction (FGR; historically referred to as IUGR) and supports a pragmatic definition based on the idea that a baby is considered ‘small’ or ‘restricted’ if its weight or abdominal circumference is below the 10th percentile—that is, if it is smaller than 90 per cent of babies at the same gestational age. FGR, considered a clinical condition, is typically multifactorial, as it can result from genetic disorders, congenital infections, teratogenic exposures, multiple pregnancies, structural abnormalities of the umbilical cord or placenta, and chronic maternal conditions such as obesity, diabetes, and hypertensive disorders [69]. These factors can lead to placental hypoperfusion, resulting from inflammatory microthrombosis and inadequate remodeling of the spiral arteries, causing an alteration in immune privilege at the maternal-fetal interface. This, combined with an intrauterine inflammatory trajectory, means that the longitudinal profile of biomarkers in preterm infants with growth restriction shows sustained inflammatory activation from birth through discharge from the neonatal intensive care unit, suggesting that inflammatory programming is already established before delivery, rather than being an exclusively postnatal phenomenon. In chronic disease settings, impaired dNK/Treg function and reduced HLA-G signaling remain plausible upstream drivers that could feed into this malperfusion–inflammation axis [70].

### 4.2. Preterm Birth and Low Birth Weight

Certain chronic maternal diseases are consistently linked to higher risks of preterm birth and low birth weight. Hypertensive disease, for example, can alter the course of pregnancy dramatically: when preeclampsia or gestational hypertension develops, reduced placental perfusion, endothelial injury, and angiogenic imbalance can erode placental function and push the pregnancy toward earlier delivery. Across autoimmune and immune-mediated conditions, a similar pattern emerges; preterm birth risk is repeatedly higher than in pregnancies without these diagnoses. Conceptually, the strongest signals have been reported for type 1 diabetes (Odds Ratio [OR] 4.36, 95% Confidence Interval [CI] 3.72–5.12) and systemic lupus erythematosus (OR 2.79, 95% CI 2.07–3.77), with more modest but still significant associations observed in Sjögren’s syndrome (RR 2.27, 95% CI 1.46–3.52), coeliac disease (OR 1.30, 95% CI 1.13–1.50), inflammatory bowel disease (OR 1.81, 95% CI 1.37–2.38), psoriatic disease (psoriasis OR 1.22, 95% CI 1.04–1.42; psoriatic arthritis OR 1.48, 95% CI 1.24–1.78), rheumatoid arthritis (OR 1.57, 95% CI 1.00–2.48), systemic sclerosis (OR 2.40, 95% CI 1.14–4.86), and thyroid autoimmunity (overall OR 1.88, 95% CI 1.32–2.67; anti-TPO positivity OR 1.33, 95% CI 1.15–1.56). Collectively, these data suggest that chronic immune dysregulation, whether systemic or organ-specific, can meaningfully shift pregnancy toward earlier delivery compared with the general obstetric population [71].

A sustained proinflammatory state, along with endothelial and decidual stress, can accelerate cervical ripening and promote myometrial activation, shortening the duration of pregnancy while simultaneously limiting fetal growth. Conditions such as obesity, hypertension, and autoimmune diseases are associated with increasingly high rates of preterm birth and low birth weight due to the pro-inflammatory state, supporting the existence of immune-mediated pathways independent of the specific disease [72]. Recent data indicate that women who begin pregnancy with multiple chronic conditions are more likely to give birth prematurely than those without chronic diseases, suggesting that multimorbidity—rather than any specific diagnosis—may influence the duration of pregnancy (Table 1). Multimorbidity is often associated with persistent low-grade inflammation, vascular and metabolic stress, and reduced physiological reserve; all of these factors can affect placental function and tip the balance toward preterm birth, whether through spontaneous mechanisms or via a medically indicated intervention when maternal-fetal compromise arises [73].

### 4.3. Congenital Anomalies

From a perinatal genetics perspective, the teratogenic burden of chronic disease is most apparent in structural malformations. Population-based analyses indicate that pregestational diabetes substantially increases the risk of congenital heart disease, while maternal overweight/obesity independently raises the odds of major defects, with risk gradients that track with increasing BMI. Notably, recent work distinguishes the risk architecture of type 1 diabetes from that of overweight/obesity, implying partially distinct teratogenic mechanisms (e.g., glucotoxicity and oxidative stress versus inflammatory pathways and folate-related metabolic effects) [74].

In a large population-based meta-analysis covering more than 80 million births and focusing on pregestational diabetes, the authors conclude that this condition is associated with a higher risk of congenital anomalies and heart disease in children [75]. Pregestational diabetes is among the chronic maternal conditions most strongly associated with congenital disease. Recent meta-analytic evidence reports odds ratios around 3.3 for congenital heart defects overall, with higher pooled estimates for specific cardiac phenotypes, including conotruncal defects, endocardial cushion defects, isolated septal defects, and left- and right-ventricular outflow tract obstruction [76].

Beyond congenital heart defects, poorly controlled pregestational diabetes—especially when hyperglycemia persists around the time of conception and during early organogenesis—is closely associated with a broader spectrum of serious congenital anomalies, including neural tube defects, caudal regression (sacral agenesis), renal and urinary tract malformations, gastrointestinal defects (anorectal or intestinal anomalies), and limb or skeletal anomalies, also known as diabetic embryopathy [77]. Chronic maternal autoimmunity leaves a congenital mark on the newborn, which is mediated during fetal development by transplacental antibodies such as anti-Ro/SSA and anti-La/SSB, which can cause neonatal lupus and congenital heart block. The most consequential manifestation is cardiac—neonatal lupus is described as the leading cause of severe congenital atrioventricular block in the neonatal period, accounting for roughly 80–95% of cases diagnosed before 28 days of life—linking maternal chronic autoimmunity to a specific congenital conduction defect with long-term implications (Table 1) [78].

**Table 1:** Effects of Maternal Chronic Conditions on Progeny.

Immediate Outcome in Progeny	Placental/Immune Pathway (Mechanistic Link)	Typical Maternal Chronic Conditions
Fetal growth restriction (FGR/IUGR) [67,79,80,81]	Chronic maternal inflammation and disrupted maternal–fetal immune tolerance promote villous/decidual inflammation, altered macrophage phenotypes (decidual macrophages; Hofbauer cells), impaired spiral artery remodeling and uteroplacental perfusion → reduced nutrient/oxygen transfer and growth restriction.	Preeclampsia/HTN; obesity; diabetes/GDM; autoimmune/inflammatory disease
Preterm birth [79,82,83]	Inflammatory cytokine signaling and placental stress can activate parturition pathways; severe placental disease (e.g., PE) often leads to iatrogenic preterm delivery to prevent maternal/fetal deterioration.	Preeclampsia; obesity; diabetes/GDM; chronic kidney/cardiometabolic disease
	Shared biology with FGR and/or prematurity: placental inflammation/vascular dysfunction limits growth; earlier delivery truncates fetal growth trajectory.	Preeclampsia; obesity; diabetes/GDM
Congenital anomalies (major structural defects) [84,85]	Maternal metabolic dysregulation and inflammatory milieu during organogenesis may increase risk; placental factors may mediate associations between maternal disease (e.g., PE) and specific anomalies (e.g., congenital heart disease).	Diabetes/GDM; obesity; hypertensive disorders/PE

Exposure to intrauterine stress leads to altered fetal programming, inducing molecular and physiological changes in developing systems—an idea now known as the Developmental Origins of Health and Disease (DOHaD) hypothesis. Recently, findings have shown that alterations in the immune system during the prenatal period are linked to the development of various diseases, such as neurodevelopmental disorders, allergic diseases, and type I diabetes in offspring [86,87,88]. Most studies focus on placental immunological disruption caused by viral or bacterial infections. Samhita et al. reported that maternal immune activation (MIA) resulting from conditions such as urinary tract infections, chorioamnionitis, diabetes mellitus, systemic lupus erythematosus, and ulcerative colitis was associated with adverse neurological outcomes in offspring during the first year of life. MIA is characterized by altered levels of proinflammatory cytokines, immune cells, and antibodies. Their findings indicated that maternal immune activation during pregnancy is linked to increased neurological morbidity in infants [87]. Experimental models support these observations. Rats exposed prenatally to immune activators such as lipopolysaccharide (LPS) show decreased dendritic arborization in neurons of the medial prefrontal cortex (mPFC) and the CA1 region of the hippocampus. Additionally, during the prepubertal period (postnatal days 10–35), reduced dendritic length in the mPFC has been reported, although these values return to control levels by day 60 [88]. Prenatal LPS exposure also reduces dentate gyrus cell proliferation and survival in 14-day-old offspring [89]. Together, these findings suggest that disruptions in neural circuit development may contribute to deficits in PFC and hippocampus-dependent tasks, such as object recognition, spatial learning, and working memory. These structural alterations have also been described in animal models of schizophrenia and in patients with the disorder (Figure 3).

## 5. Therapeutic and Preventive Perspectives

Current treatments for chronic diseases rely on nutritional and lifestyle interventions, along with pharmacological therapy for each condition. The lifestyle management requirements for GDM and obese patients include an adequate food calorie intake for glycemic goals for fetal and maternal health, physical activity, and weight management [90]. When lifestyle changes alone are insufficient to regulate the disease, the usual treatment for GDM is insulin, metformin, and/or glyburide therapy [90]. In an in vitro model that simulates the low-grade sterile chronic inflammation observed in placental tissue during gestational diabetes mellitus, insulin and metformin treatment reduced IL-1β and IL-6 cytokine levels. Moreover, under hyperglycemic conditions, when the explants were challenged with E. coli, these treatments attenuated bacterial invasion [91]. Although metformin and glyburide, administered alone or in combination, improve β-cell responsivity [92], they are not recommended as first-line therapy since both drugs can cross the placenta [90].

The American College of Obstetricians and Gynecologists recommends that women with a medical history of hypertensive disorders, such as early-onset preeclampsia (PE), begin low-dose aspirin therapy [93]. Indeed, previous studies have demonstrated that oral aspirin intake reduces the risk of PE and preterm birth [94,95]. The proposed mechanisms involved regulating the proportion of thromboxane A2/prostacyclin [96], reducing hypoxia-induced soluble fms-like tyrosine kinase 1 (sFlt1) production via c-Jun NH2-terminal kinase/activator protein-1 (JNK/AP-1) [97], and inhibiting cyclo-oxygenase 1 (COX1) [98,99]. In addition, aspirin treatment restored the endothelial nitric oxide synthase (eNOS) by reducing MIR155HG expression. It improved the impaired relaxation responses induced by TNF-α treatment in human placental arterial vessels [100]. It also decreased proinflammatory cytokine production (INF-γ and IL-8) in mesenchymal stem/stromal cells derived from term PE patients [101]. Interestingly, in a lipopolysaccharide-induced preeclampsia-like animal model, aspirin attenuated the increased blood pressure, proteinuria, and expression of TNF-α, IL-6, and IL-1β in placental tissue, potentially via the TLR2 and TLR4, MyD88, and NF-κB signaling pathways [102]. These findings suggest that aspirin treatment could be effective for hypertensive disorders of pregnancy. However, the optimal timing and dosage for aspirin administration remain controversial [94,95]. Furthermore, as a first-line therapy for non-severe hypertensive disorders, methyldopa and labetalol administration is also considered, and as a second-line therapy, nifedipine [103]. The use of antihypertensive drugs is well documented by their vascular effects that contribute to lower blood pressure. However, data remain scarce regarding how these pharmacos may influence the regulation of placental immunological privilege in hypertensive disorders. A preclinical study demonstrated that methyldopa administration reduced VEGF, TGF-β, and hypoxia-inducible factor 1 (HIF-1α) in the placental tissue from pregnant spontaneously hypertensive rats, accompanied by attenuated systolic blood pressure [104]. It is worth noting that there is limited evidence demonstrating that these treatments do not impair uteroplacental function [105,106,107,108].

## 6. Future Directions and Conclusions

The studies presented here provide evidence of changes in placental immune response, shifting from a tolerogenic environment toward a pro-inflammatory one during gestation when a chronic disease is present or develops during pregnancy itself. The inflammation triggered in these diseases is termed meta-inflammation, characterized by a low chronic state of inflammation mediated by activation of DAMPs’ receptors. This environment turns a local stress response into a loop of amplifying cellular damage and inflammation that drives organ dysfunction. Further, clinical evidence suggests that these maternal chronic conditions are related to fetal outcomes, including preterm birth, FGR, CHD, etc.

Despite significant advances in our understanding of placental immunology, there are still several gaps in our knowledge that limit the development of specific interventions for pregnancy-related disorders. Moreover, longitudinal human studies and refined animal models are required to elucidate how disturbances in placental immunological privilege in pregnancy disorders could contribute to the Developmental Origins of Health and Disease (DOHaD). Clinical treatments for various chronic conditions of pregnancy, such as diabetes, obesity, and gestational hypertension, continue to rely on metabolic or vascular interventions—such as aspirin, antihypertensive medications, or metformin—which affect the function of immune cells in the decidua. Therefore, it is important to identify immunological biomarkers that enable the early detection of immune dysregulation before clinical symptoms manifest, thereby allowing for the development of safer, naturally derived therapeutic interventions aimed at reducing the rate of preterm births.

## Figures and Tables

**Figure 1:**
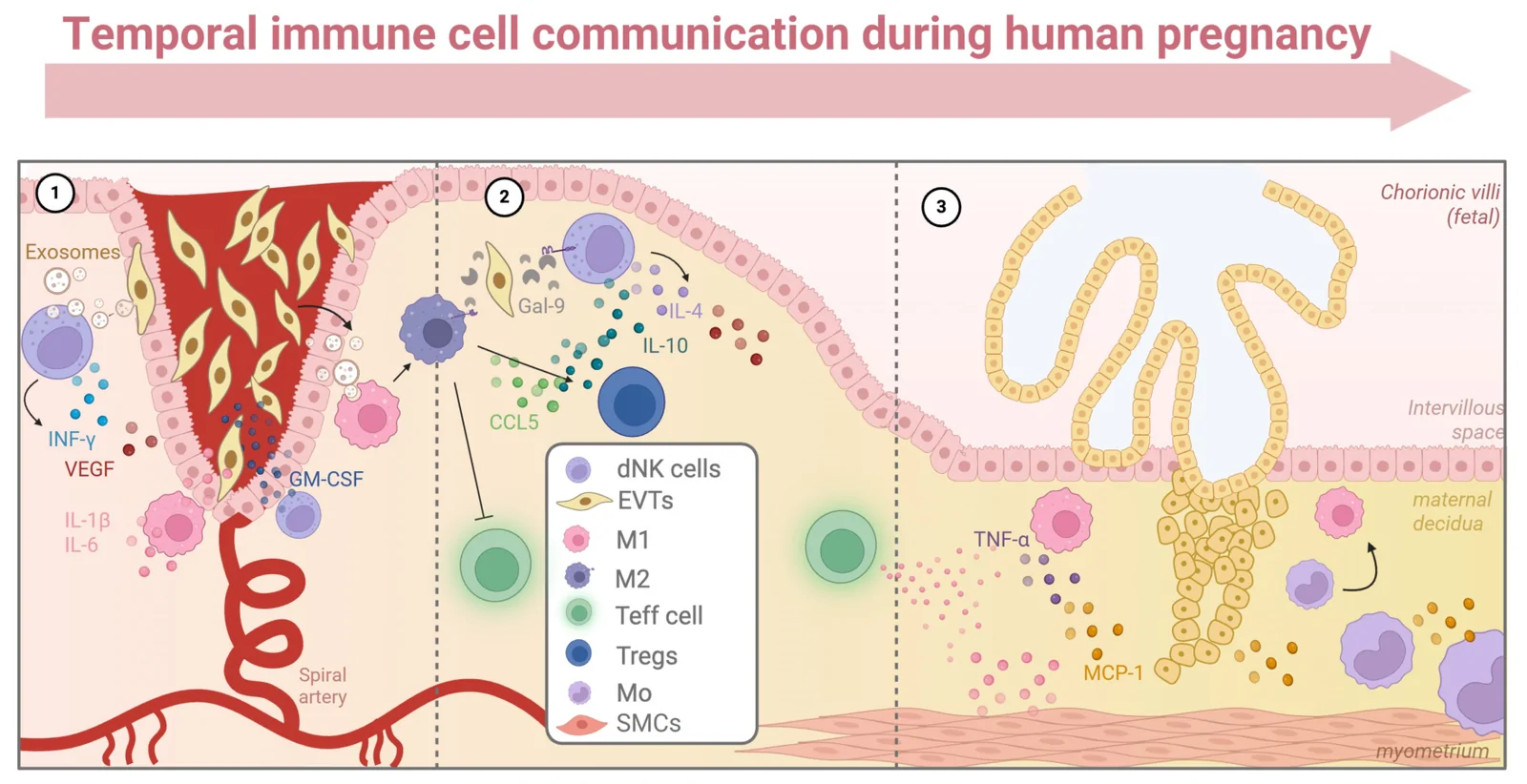
Overview of immune cell crosstalk at the maternal-fetal interface during gestation—schematic representation of immune cell communication during human pregnancy. The illustration shows the transition of placentation in early pregnancy, characterized by (1) an initial inflammatory environment that facilitates vascular remodeling, (2) the establishment of immune tolerance during the second trimester, and (3) proinflammatory signals at the end of pregnancy; immune cells interact with SMCs and promote the labor process. dNK cells: decidual natural killer cells, EVTs: extravillous trophoblasts, M1: macrophages M1, M2: macrophages M2, Teff cells: T effector cells, Tregs: regulatory T cells, Mo: monocytes, SMCs: smooth muscle cells. (Figure created in https://BioRender.com). Created in BioRender. Castro, D. (2026) https://BioRender.com/kzgij2j, accessed on 10 August 2026.

**Figure 2:**
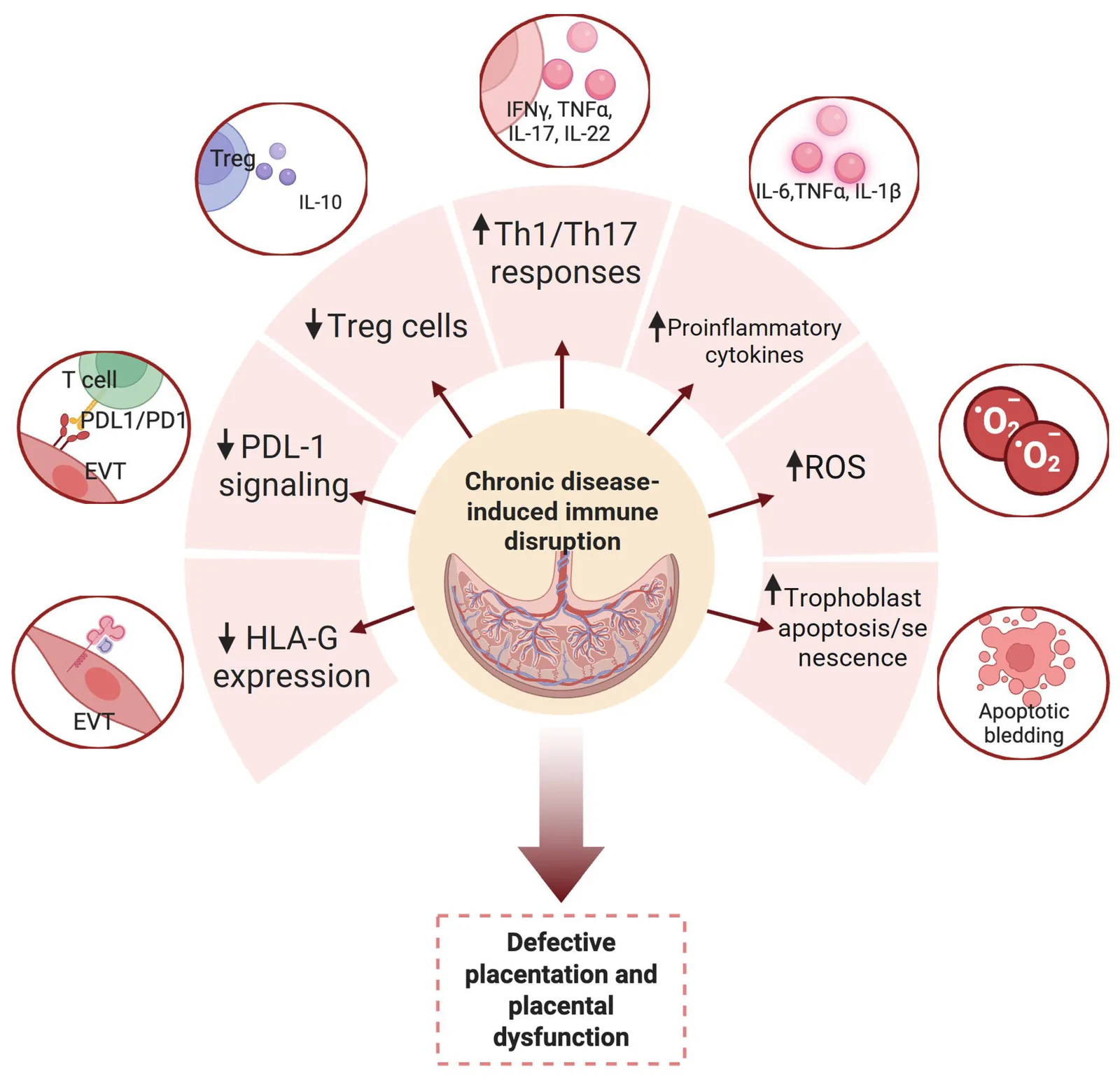
Chronic Diseases as Disruptors of Placental Immune Privilege. Maternal conditions during pregnancy induce immune dysregulation, characterized by the downregulation of molecules essential for recognizing fetal cells and impaired tolerance mechanisms. Consequently, the exacerbation of proinflammatory responses and oxidative stress leads to placental dysfunction. (Figure created in https://BioRender.com). Created in BioRender. Castro, D. (2026) https://BioRender.com/kzgij2j, accessed on 10 August 2026.

**Figure 3:**
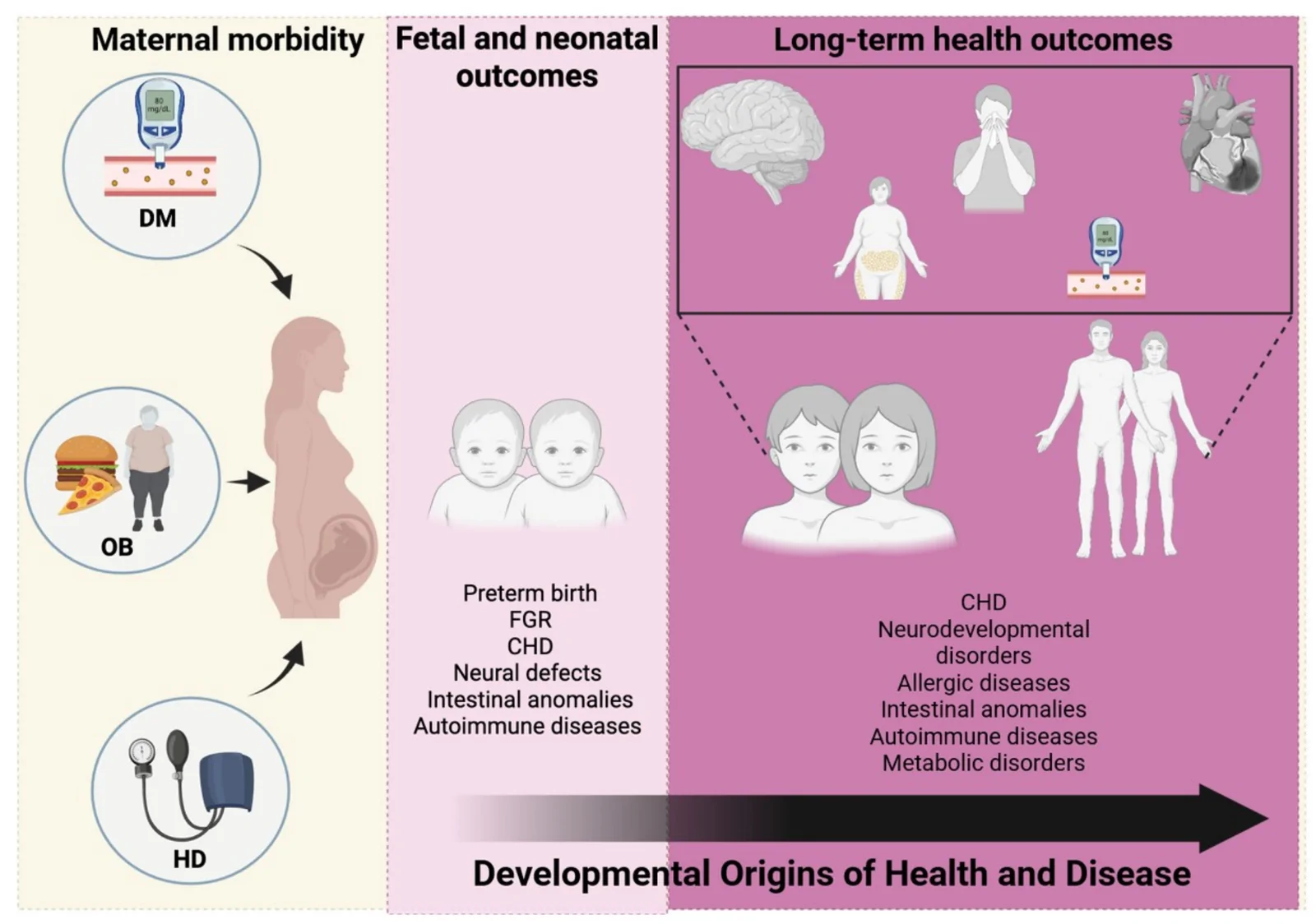
Impact of maternal chronic conditions on offspring across the life course. Maternal chronic conditions, including Diabetes Mellitus (DM), Obesity (OB), and hypertensive disorders (HD), contribute to fetal, neonatal, and adulthood outcomes. (Figure created in https://BioRender.com). Created in BioRender. Castro, D. (2026) https://BioRender.com/kzgij2j, accessed on 10 August 2026.

## Data Availability

No new data were created or analyzed in this study. Data sharing is not applicable to this article.

## Abbreviations

The following abbreviations are used in this manuscript:

CCL	CC-motif chemokine ligand
CCR2	C-C chemokine receptor type 2
CHD	congenital heart defects
CI	confidence interval
CTLA4	T-lymphocyte-associated protein-4
DAMPs	damage-associated molecular patterns
DCs	dendritic cells
DOHaD	Developmental Origins of Health and Disease
dNK	decidual natural killer
eNOS	nitric oxide synthase
EVTs	extravillous trophoblasts
FGR	fetal growth restriction
Gal-9	Galectin-9
GDM	gestational diabetes mellitus
GM-CSF	granulocyte-macrophage colony-stimulating factor
HIF-1α	hypoxic inducible factor 1
HLA	Human Leukocyte Antigen
INF-γ	interferon gamma
IL-4	interleukin-4
IL-6	interleukin-6
IL-10	interleukin-10
ILCs	innate lymphoid cells
KIRs	via killer immunoglobulin-like receptors
LPS	lipopolysaccharide
MCP-1	monocyte chemotactic protein-1
MIA	maternal immune activation
MIP	macrophage inflammatory protein
Mo	monocytes
mPFC	medial prefrontal cortex
Mϕs	macrophages
NFKB1	Nuclear Factor Kappa B Subunit 1
OR	odds ratio
pbNK	peripheral blood natural Killer
PE	preeclampsia
PD1/PDL-1	programmed cell death-1/programmed cell death ligand-1
ROS	reactive oxygen species
sFlt-1	soluble fms-like tyrosine kinase 1
SMCs	smooth muscle cells
Tim-3	mucin domain-containing molecule-3
TGF-β	Transforming Growth Factor beta
TLRs	Toll-like receptors
TNF-α	Tumor Necrosis Factor alpha
Treg	regulatory T cell
uNK	uterine natural killer
VEGF	vascular endothelial growth factor
